# Stem Cell Impairment at the Host-Microbiota Interface in Colorectal Cancer

**DOI:** 10.3390/cancers13050996

**Published:** 2021-02-27

**Authors:** Marinella Marzano, Bruno Fosso, Elisabetta Piancone, Giuseppe Defazio, Graziano Pesole, Mariangela De Robertis

**Affiliations:** 1 Institute of Biomembranes, Bioenergetics and Molecular Biotechnologies, Consiglio Nazionale delle Ricerche, 70126 Bari, Italy; m.marzano@ibiom.cnr.it (M.M.); b.fosso@ibiom.cnr.it (B.F.); graziano.pesole@uniba.it (G.P.); 2 Department of Biosciences, Biotechnology and Biopharmaceutics, University of Bari ‘Aldo Moro’, 70126 Bari, Italy; elisabetta.piancone@uniba.it (E.P.); giuseppe.defazio@uniba.it (G.D.)

**Keywords:** cancer stem cells, microbiome, colorectal cancer, OMICS technologies, precision medicine

## Abstract

**Simple Summary:**

The mechanisms underlying the effects of exogenous factors on impaired intestinal stem niche homeostasis in colorectal cancer pathogenesis are an important and ongoing focus for stem cell research. The most recent findings indicate that dysbiosis (changes in the homeostatic gut microbiota composition) can induce an aberrant reprogramming of the intestinal stem cells (ISCs) through several mechanisms, such as impaired metabolism and abnormal activation of the immune system, as well as genetic and epigenetic instability. The review goes beyond the discussion of the involvement of gut dysbiosis in colorectal cancer development, mainly summarizing the most recent findings linking the gut microbiome to colorectal cancer pathogenesis through the ISC niche impairment. The most significant advances in this field are described, focusing on different “omics” strategies, with a particular interest for the multiomics approach which will be gradually included into the framework of precision medicine.

**Abstract:**

Colorectal cancer (CRC) initiation is believed to result from the conversion of normal intestinal stem cells (ISCs) into cancer stem cells (CSCs), also known as tumor-initiating cells (TICs). Hence, CRC evolves through the multiple acquisition of well-established genetic and epigenetic alterations with an adenoma-carcinoma sequence progression. Unlike other stem cells elsewhere in the body, ISCs cohabit with the intestinal microbiota, which consists of a diverse community of microorganisms, including bacteria, fungi, and viruses. The gut microbiota communicates closely with ISCs and mounting evidence suggests that there is significant crosstalk between host and microbiota at the ISC niche level. Metagenomic analyses have demonstrated that the host-microbiota mutually beneficial symbiosis existing under physiologic conditions is lost during a state of pathological microbial imbalance due to the alteration of microbiota composition (dysbiosis) and/or the genetic susceptibility of the host. The complex interaction between CRC and microbiota is at the forefront of the current CRC research, and there is growing attention on a possible role of the gut microbiome in the pathogenesis of CRC through ISC niche impairment. Here we primarily review the most recent findings on the molecular mechanism underlying the complex interplay between gut microbiota and ISCs, revealing a possible key role of microbiota in the aberrant reprogramming of CSCs in the initiation of CRC. We also discuss recent advances in OMICS approaches and single-cell analyses to explore the relationship between gut microbiota and ISC/CSC niche biology leading to a desirable implementation of the current precision medicine approaches.

## 1. Introduction

Intestinal stem cells (ISCs) are a population of rare undifferentiated cells located in the intestinal crypts, responsible for tissue homeostasis and regeneration after injury or inflammation. ISCs play a fundamental role in maintaining the mucosal barrier through continuous and regulated proliferation, which results in the constant replacement of the intestinal epithelium. This mechanism preserves the intestinal barrier function and prevents the pathogen invasion and physical insults, which activate a chronic or acute inflammation in both pathologic or physiologic conditions [1]. 

The alteration of ISCs proliferation mechanisms is intimately involved in intestinal diseases, including Intestinal Bowel Disease (IBD) and colorectal cancer (CRC) [2]. In particular, many studies have focused on the mechanisms underlying CRC initiation through the conversion of ISCs into cancer stem cells (CSCs), also known as tumor-initiating cells (TICs) [3], opening a new avenue to identify new CSCs-target therapies for CRC. However, it has become clear that the insight into the intestinal crypt prior to the establishment of a CSC niche is an exciting research area that has much more to reveal.

The microenvironmental context is critical to reprogramming ISC niches as they are located at the host-microbiota interface. Indeed, there is a complex interplay between the epithelial barrier, its microbial ecosystem, and the local immune system [4]. Under normal conditions, the intestinal epithelium interacts with a varied community of microorganisms, including bacteria, fungi, and viruses, which are considered to be the intestinal microbiota [5]. The large diversity within the gut microbiota is finely balanced to protect the intestinal mucosal barrier. For example, epithelial cells recognize and communicate with the resident microbiota via pattern-recognition receptors (PRRs), including Toll-like receptors (TLRs), present on both intestinal differentiated epithelial cells and ISCs [2]. Unlike other stem cells, ISCs coexist indeed with the intestinal microbiota. For example, species belonging to the genus *Lactobacillus* can directly or indirectly affect ISCs proliferation and differentiation. Conversely, ISCs protect themselves from butyrate produced by beneficial microbes residing in the intestinal lumen [5,6].

Recent evidence has shown that disruption of the normal homeostatic balance between the host’s mucosal cells and the gut microbiota results in aberrant immune responses against resident commensals, leading to chronic inflammation and ultimately predisposing the patient to CRC [7]. In this proinflammatory state, ISCs directly sense and respond to microbiota [8].

The mechanisms through which exogenous factors, such as the gut microbiota alteration, confer their effects on the ISC niche have become an exciting but complex and controversial field of research focusing on stem cell biology. Here, we summarize the most up-date evidence regarding the molecular mechanisms and metabolic processes underlying the complex interaction between intestinal microbiota and ISCs, which is crucial for determining ISCs homeostatic behavior and aberrant reprogramming in CRC initiation. Furthermore, the review considers the advances in research on the OMICS technologies applied to both preclinical and clinical studies to explore the molecular mechanisms of CRC-related imbalance in the intestinal microbiota-ISCs relationship.

## 2. ISC Niche Structure and Functional Organization

The intestinal epithelium is organized into villi, which are finger-shape protrusions projected into the lumen of gut—with the exception of the colon—and crypts of Lieberkühn, often referred to simply as “crypts”, which are pits found between the villi where the ISCs reside. Through finely regulated proliferation, the ISCs play an essential role in the intestinal homeostasis. 

ISCs division usually produces daughter cells that are a new stem cell and a transient-amplifying cell (TAC). After additional 4–5 divisions, TACs differentiate into intestinal epithelial cell subtypes, including enterocytes (absorptive), goblet cells (mucus producing), neuroendocrine cells and Paneth cells (mucosal defense effectors) [9]. Aside from Paneth cells, all these cells migrate to the tip of the villi along the crypt-villus axis during the differentiation process. All the cells of the intestinal epithelium reach the top of the villus in 3–5 days and become competent in the digestion and absorption of dietary nutrients. In this position, the cells go through programmed cell death and are shed into the lumen (about 15 billion cells per day). Since these cells are highly exposed to many luminal pathogens or chemicals that pass through the intestinal lumen, their rapid turnover is likely to be important in limiting the amount of potentially damaged epithelial cells. To avoid a subsequent breakdown of the epithelial barrier, it is essential that new epithelial cells replace the lost cells. This mechanism is made possible by the ISCs compartment. Indeed, ISCs proliferation is essential for continuously contributing to fueling the entire intestinal epithelium with newly generated epithelial cells.

Since ISCs routinely undergo asymmetric division, it makes sense that there are at least two types of ISCs in the bottom of the niche, known as crypt basal columnar (CBC): cells that express Leucine-rich repeat-containing G-protein coupled receptor 5 (Lrg5) and slow-cycling cells that are B lymphoma Mo-MLV insertion region 1 homolog (Bmi1) positive [10].

CBC Lgr5+ ISCs are actively cycling and rapid proliferating ISCs, as demonstrated by the use of the lineage tracing technology [11]. In the crypt region these cells are found scattered among Paneth cells where they continuously regenerate epithelial cells which migrate along the crypt-villus axis [12]. LGR5 is a target of the Wnt signaling pathway and is the most consensual ISC marker to date [13]. Normally, CBC Lgr5+ ISCs can self-renew through a symmetrical division into either two CBCs or two TACs [14]. In addition, ISCs may originate from a possible dedifferentiation of TACs or Paneth cells into Lgr5+ cells in response to Wnt3A or irradiation [15]. Interestingly, it was also revealed that a pool of Lgr5+ cells expressing the RNA-binding protein Mex3a are crucial for maintaining the Lgr5+ ISC pool in case of injury [16].

Bmi1+ ISCs are slow-cycling cells in the 4+ cell position of the crypt (four cells away from the base of the crypt), which are quiescent and express two peculiar stem cell markers, namely Bmi1 and mTert [17,18]. Quiescent Bmi1+ ISCs form a reserve ISCs, which is important for facilitating epithelial regeneration after injury. In contrast to Lgr5+ cells, which are more important for homeostatic functions, reserve ISCs are defined as the injury-inducible reserve ISCs population [10].

Several studies, mostly including lineage tracing experiments, have allowed researchers to identify novel ISCs markers through direct overlap with the expression of Lgr5 or Bmi1 in actively-cycling ISCs or reserve ISCs, respectively. As extensively reviewed elsewhere [19], ISC markers can be distinguished as (i) “active ISC” markers, such as Lgr5, Olfactomedin-4 (Olfm4), SPARC related modular calcium binding 2 (Smoc2), Achaete scutelike 2 (Ascl2), Ring finger protein 43 (Rnf43), Zinc and ring finger 3 (Znrf3); (ii) “reserve ISC” markers, including Bmi1, leucine rich repeats and immunoglobulinlike domains 1 (Lrig1), HOP homeobox (Hopx), mouse telomerase reverse transcriptase (mTert); (iii) markers with a differential expression pattern, such as SRY-box 9 (Sox9), EPH receptor B2 (EphB2), prominin 1 (Prom1), Musashi-1 (Msi1), Mex-3 RNA-binding family member A (Mex3a), Krüppel-like factor 4 (KLF4), Doublecortin and CaMkinase-like-1 (Dclk1).

In addition to the ISC subpopulations, the ISC niche includes different cells that release factors orchestrating ISCs quiescence or proliferation/differentiation, including Paneth cells, stromal/myofibroblast, and immune cells.

Paneth cells are located at the bottom of the crypt where the CBC Lgr5+ ISCs are also located. Through paracrine signaling, they can regulate ISCs by secreting critical niche signals such as Wnt3, Epidermal growth factor (EGF), transforming growth factor-α (Tgf-α), and the Notch ligand Delta-like 4 (Dll4), ADP ribose [15,20]. In the colon, there are Paneth-like cells, which include deep crypt secretory cells expressing the regenerating islet-derived family member 4 (Reg4) marker [21]. Paneth cells and Paneth-like cells contribute to the ISC niche stem functions in a context-dependent manner. For instance, upon inflammation Paneth cells may acquire stemlike features by re-entering the cell cycle and dedifferentiating; in this way they contribute to the tissue regenerative response [22]. This suggests that Paneth cells disruption may be at the origin of IBD-related intestinal tumors, parallel to what observed in the adenomatous polyposis coli (Apc)-driven intestinal tumorigenesis [23].

Myofibroblasts are a second important class of cells located around the crypts, which are critical for maintaining the proliferative status and turnover of ISCs [24]. They form the essential Wnt-secreting niche for colon stem cells [25,26] and release some key bioactive factors, such as R-Spondin1, which enhances Wnt3-activated signals, and Noggin, which is an antagonist of BMP/Smad signaling pathway [24,27].

Finally, various immune cells contained in the intestinal lamina propria contribute to the regulation of ISCs by secreting immunomodulators, including cytokines, capable of acting directly or indirectly on ISCs proliferation. Among the secreted cytokines, IL-6 and IL-17 have been shown to stimulate ISC proliferation [28]. Moreover, Jeffrey et al. demonstrated that the inhibition of IL-6 signaling by a neutralizing antibody can prevent ISC proliferation in an inflammatory context and suggested that autocrine IL-6 signaling in the gut epithelium affects crypt homeostasis via the Paneth cells and the Wnt signaling pathway [29].

## 3. Dysfunction of ISC Niche in CRC

The stem cell niche is a specialized microenvironment which regulates ISCs’ specific properties by directing a complex network of stem cell-regulatory pathways, including Wnt, Notch, bone morphogenetic protein (BMP), Hedgehog (Hh), EGFR/MAPK, and Eph/Ephrin [30]. By virtue of being stem cells in the gut, the role of ISCs as the “cell-of-origin” of CRC was examined. The long-term clonogenic potential of ISCs, required for intestinal epithelium regeneration, exposes the ISC compartment to a higher risk of accumulation of DNA mutations, thus making ISCs ideal candidates for tumor initiation [31]. Furthermore, ISCs constitute a long-lived cellular compartment, unlike differentiated cells, which are rapidly exfoliated into the gut lumen and are consequently limited to clonally expand.

In recent years, the research has clearly established that the malignant transformation of normal ISCs, and more rarely the dedifferentiation of mature intestinal cells, can give rise to CSCs responsible for cancer development and propagation [32]. As suggested by the “CSCs model”, this small subset of cancer cells not only maintains the self-renewal capacity required to initiate and sustain tumor growth, but also has a differentiation potential through which to generate a range of heterogeneous cancer cell types within the tumor, according to a differentiation hierarchy [33]. However, it should be emphasized that in some cases TACs can be responsible for the onset of adenoma, albeit as efficiently as stem cells, and that differentiated cells can also initiate tumorigenesis but only in presence of additional events such as inflammation or changes in the microenvironment [34]. Therefore, while ISCs are not the only possible cells of origin for CRC, they are certainly the most potent cells for tumor initiation.

A dysregulation of the self-renewal and pluripotency signaling pathways occurs in the ISC compartment through several genetic and epigenetic changes, leading to cell transformation and ultimately to the generation of CSCs. Over the last few decades, multiple markers have been identified to reveal the CSC subpopulation in CRC distinguishing between cell surface markers, including Prominin-1 (CD133), CD44, CD166, EpCAM, EphB2, Lgr5, D26, CD44v6, and intracellular proteins, such as aldehyde dehydrogenase 1 (ALDH1), Bmi1, Musashi-1 (MSI-1) and doublecortinlike kinase 1 (DCLK1) [35,36].

From a pathogenetic point of view, the main driver and tumor-suppressor genes have been well defined in the intestinal epithelial cells, including APC, the KRAS oncogene, and TP53 [37]. Recently, it has been revealed that mutations in these genes significantly influence the clonal behavior of ISCs [38,39]. A common early oncogenic event in CRC is the mutation within the APC gene [38], which results in ineffective β-catenin degradation and induces a constitutively active Wnt pathway. When the ISC compartment harbors APC mutations, it undergoes an uncontrolled expansion followed by adenoma formation, as also confirmed in the mouse genetic models where adenomas appeared only in case of APC mutations targeting to ISCs rather than differentiated cells [40]. In fact, when an ISC acquires an APC mutation, it gains a higher probability of niche fixation than nonmutated ISCs [38]. Interestingly, a much higher fission rate has been observed in KRAS mutant crypts [39], while P53 mutations have a higher niche fixation rate only in the presence of colitis [38]. This highlights the importance of extrinsic factors in the transformation of intestinal cells. Another key factor involved in the ISCs transformation is the antiapoptotic B-cell lymphoma 2 (Bcl-2) protein, which is particularly expressed in Lgr5+ CBCs. Bcl-2 is also an important target gene of the nuclear factor (NF)-κB pathway, which influences full adenomatous outgrowth by also acting on the differentiated compartment in combination with the constitutive active Wnt signaling [41].

It could be speculated that inflammatory signals from the intestinal environment may stimulate the oncogenic potential of ISCs by inducing extensive cellular plasticity due to the activation of the tissue injury/repair mechanisms. Indeed, during inflammation, characterized by a rapid regeneration of the intestinal epithelium, ISCs can undergo inappropriate proliferation and lead to the expansion of the pool of cells prone to malignant transformation [42].

## 4. Effects of Intestinal Microbiota on ISCs Homeostasis 

The distal part of the small intestine and colon is the habitat of a densely populated ecosystem of commensal microbiota, which lives in a mutually beneficial state with the host [43]. Many recent studies have analyzed the principal characteristics of the intestinal microbiota, which is composed of bacteria, archaea and fungi. Thanks to phylogenetic analysis, a matching bacterial content was found in the distal ileum, ascending colon and rectum [44], with the highest microbiota content in the distal ileum and colon (10^12^–10^14^ bacterial cells/mL of luminal content) [45].

Although there is a symbiotic relationship between the microbiota and the host, the close proximity of the microbiota to the intestinal wall poses risks of invasion with consequent health problems. The intestinal epithelium therefore plays a crucial role in maintaining not only tissue homeostasis but also a strategic compartmentalization between the lumen and the host, acting as a continuous physical barrier against the intestinal microbes, toxins, dietary products, and inorganic materials [46]. The endothelium also forms a gut-vascular barrier (GVB) that controls the translocation of antigens into the bloodstream and prohibits the entry of the microbiota [47]. 

In addition, the intestinal epithelial barrier is composed of a mucus layer, which allows for an additional barrier against the luminal content. The mucus structure differs between the small intestine, where it consists of a single layer 100–250 μm thick, and the colon, where there are two distinct layers reaching 700 μm in thickness. In the colon, the inner mucus layer is firmly attached to the epithelium and is normally not permeable to bacteria, while the outer and more voluminous layer is slightly attached to the inner layer and forms a habitat for large numbers of bacteria [48]. Mucus is mainly composed of mucin glycoproteins, with Muc2 being the predominant component of both mucus layers. Mucins are secreted by goblet cells or reside as membrane-bound proteins on the apical surface of the epithelium. In addition, goblet cells secrete various bioactive molecules such as trefoil factor peptides (Tff), resistinlike molecule β (Relmβ), and Fc-γ binding protein [49], which are involved in gastrointestinal defense and repair by promoting epithelial restitution. In a concerted way, the Paneth cells provide a range of antimicrobial peptides, including α-defensins, angiogenin-4, lysozyme and secretory phospholipase A2 (Pla2), that function in host defense and in establishing and maintaining the intestinal microbiota [50]. 

Moreover, adaptive immune mechanisms have widely evolved as a means of contributing to the intestinal epithelial barrier. Among them, secreted immunoglobulins A (sIgAs) specific for antigens derived from commensal intestinal bacteria are produced by Lipopeptide/lipoprotein (LP) plasma cells [51]. Through binding to the polymeric immunoglobulin receptor (pIgR), which is expressed exclusively on the apical membrane of intestinal epithelial cells, sIgA can transcytose through epithelial cells to be secreted into the gut lumen. There, IgAs influence commensal gene expression [52], gut metabolic homeostasis and the immune ecosystem [53,54] and prevent microbial translocation across the epithelial barrier [55]. More generally, the immune system is critical in maintaining a mutualistic host-microbiota relationship [56,57]. Several types of immune cells dispersed in the subepithelial lamina propria, such as B and T cells, antigen presenting cells, namely dendritic cells (DCs), and macrophages, take part to the elimination of commensals that translocate across the intestinal epithelial cell barrier [58]. Notably, the maturation of the gut immune system depends on the microbiota and the composition of the gut microbiota, in turn, plays a fundamental role in regulating the activation of the immune system in the intestine, for example by conditioning both pro- and anti-inflammatory T cell populations, which can be important in the pathogenesis of inflammatory and autoimmune diseases [59,60,61].

**Figure 1:**
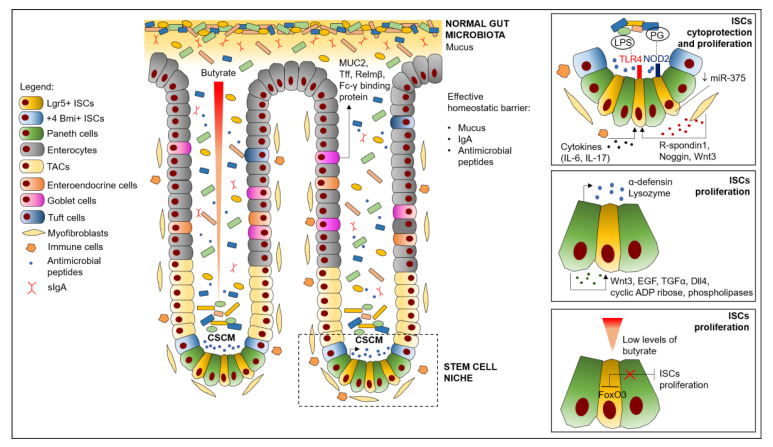
Effects of intestinal microbiota on ISCs homeostasis. Abbreviations: CSCM, crypt-specific core microbiota; Dll4, Notch ligand Delta-like 4; EGF, epidermal growth factor; FoxO3, Forkhead box protein; ISCs, intestinal stem cells; LPS, lipopolysaccharide; MUC2, mucin 2; NOD2, nucleotide-binding oligomerization domains; PG, peptidoglycan; Relmβ, resistinlike molecule β; ROS, Reactive Oxygen Species, sIgA, secreted immunoglobulin A; TAC, transient-amplifying cells; Tff, trefoil factor peptides; Tgf-α, transforming growth factor-α; TLR4, Toll-like receptor 4.

Recent studies have shown that the specific microbial composition of the crypt is distinct from that of the intestinal lumen. In the colonic crypt environment, a Crypt-Specific Core Microbiota (CSCM) was found populated by members of aerobic non fermentative (such as *Proteobacteria*) and anaerobic taxa, namely *Acinetobacter*, *Stenotrophomonas*, and *Delftia* genera [8,62]. CSCM could have a protective and homeostatic role within the ISC niche by modulating stem cells’ functions through continuous stimuli, based on the expression of particular microbe-associated molecular patterns or on the production of specific metabolites, which can influence the differentiation or proliferation pathways, as illustrated in the following sections and in Figure 1.

### 4.1. ISCs Regulation Mediated by Microbiota Engagement of PRRs 

A continuous crosstalk between intestinal mucosa and microbiota occurs at the level of pattern-recognition receptors (PRRs) located in the intestinal epithelial cells. This class of transmembrane or intracytoplasmic receptors is characterized by the ability to specifically sense distinctive microbial macromolecular ligands referred to as pathogen-associated molecular patterns (PAMPs), which include lipopolysaccharide (LPS), flagellin, peptidoglycans, and formylated peptides [63]. Toll-like receptors (TLRs) and Nucleotide-binding oligomerization domainlike receptors (NLRs) are two important families of PRRs [64].

Recent studies in mouse models have revealed the involvement of different classes of TLRs in the dynamics of the intestinal crypt after LPS injection. In particular TLR4, which is expressed in the base of the murine intestinal crypt, has been found to affect ISCs apoptosis and proliferation in a manner mediated via the p53 upregulated modulator of apoptosis (Puma) but in a Myeloid differentiation primary response 88 (Myd88)-independent manner [65,66]. Naito et al. identified a TLR4-dependent program, activated by LPS, which affects crypts at different stages of epithelial differentiation [67].

Important interactions occur between TLRs and the Wnt/β-catenin signaling pathway. For example, TLR4 inhibits enterocyte proliferation by suppressing the Wnt signaling, through the downmodulation of the Wnt receptor LRP6 [68]. Moreover, Wnt5a and Wnt10b were found to have a conserved NF-κB binding site, which allows for the binding of NF-κB, a component of a major pathway downstream of TLR signaling [69].

Although the direct effect of TLR signaling on ISC functions is still largely unknown, all this evidence firmly suggests the presence of a crosstalk between the microbiota, TLR signaling, and the Wnt and Notch pathways, influencing cell proliferation and differentiation.

Other PRRs that belong to the NLRs family mentioned above, namely the nucleotide-binding oligomerization domains (NOD), such as NOD1 and NOD2, are important for ISCs homeostasis. In particular, a recent study has shown that Lgr5+ ISCs cells constitutively express higher levels of NOD2 than Paneth cells and that NOD2 activation by peptidoglycan derived from commensal and pathogenic bacteria exerts a strong cytoprotection against ISCs death mediated by oxidative stress [70]. Therefore, NOD2 triggers stem cell survival and mediates gut epithelium restitution in the presence of microbiota-derived molecules.

Taken together, these findings suggest that differential stimulation of PRRs in determining the strength of Wnt-β-catenin or Notch signaling by the microbiota or its derivatives may establish a conceptual framework for the development of novel therapeutic strategies aimed at regulating ISCs in cancer as well as in other pathological changes in crypt architecture.

### 4.2. ISCs Regulation Mediated by Microbiota Production of Reactive Oxygen Species (ROS) 

The physical interaction between the gut and intestinal microbiota rapidly induces ROS generation in gut epithelial cells, resulting in ROS-mediated stimulation of cellular proliferation and motility, as well as modulation of the innate immune signaling [71]. Redox homeostasis has been shown to critically affect stem cell differentiation and cell self-renewal [72]. Consistent with this, a recent study has revealed that members of the commensal genus *Lactobacillus* can stimulate NADPH oxidase 1 (Nox1)-dependent ROS generation and subsequent cellular proliferation in ISCs after initial ingestion in *Drosophila* and mice under physiological conditions [73]. These ROS-induced effects in ISCs are likely to be mediated by the Wnt and Notch signaling pathways [74]. Nevertheless, it is still unclear whether ROS act as direct inducers of ISCs proliferation and differentiation signaling or indirectly cause damage signals that induce epithelium regeneration involving the modulation of ISCs function.

### 4.3. ISCs Regulation Mediated by Microbiota-Derived Metabolites

Several recent studies have demonstrated the role of microbiota-derived metabolites in ISCs proliferation.

An interesting class of gut microbial metabolites is that of short-chain fatty acids (SFCA) including acetate, butyrate and propionate [75]. Of interest is butyrate, a byproduct of fiber fermentation produced by beneficial butyrogenic microbes such as *Faecalibacterium prausnitzii, Eubacterium rectale,* and *Roseburia* species, capable of inhibiting ISCs proliferation through a Forkhead box protein (FOXO)-3-dependent mechanism [6]. However, since luminal butyrate suppresses ISCs proliferation only when it reaches the crypt cells, Kaiko et al. further suggested that under normal conditions, colonocytes likely metabolize butyrate for energy and therefore prevent butyrate from reaching the ISCs [6]. 

In line with the aberrant effect on proliferation and differentiation exerted by butyrate, other SFCAs, such as propionate or acetate, also demonstrated an inhibitory effect on the differentiation capacity of human chorion-derived mesenchymal stem cells (sMSCs) [76] and multipotent adipose tissue-derived stem cells [77], respectively. However, further research is needed to indicate possible associations between these SFCA and ISCs regulation.

### 4.4. ISCs Regulation due to Microbiota Effects on Paneth Cells 

Previous studies have shown that Paneth cells’ secretory functions are important for the differentiation and proliferation of ISCs in the crypt [15]. As previously described, they also exhibit important antimicrobial function by secreting bactericidal proteins such as α-defensins and lysozyme, reportedly under IFN-γ control [78]. 

Paneth cells directly sense the gut microbiota and help maintain homeostasis at the intestinal host-microbial interface [79]. For example, *Salmonella* infection might induce a Paneth cell differentiation program—likely in the TAC as the main progenitor responder. A consecutive and time-dependent upregulation of β-catenin, EphB3, and Sox9 in these cells results in the expansion of the Paneth cell population, which is not merely a response to microbial products but reflects a targeted response to limit *Salmonella* penetration across the mucosal barrier [80].

Hirao et al. [81] demonstrated that Paneth cells are the first to respond to pathogen infections and induce gut inflammation through IL-1β signaling in the intestinal crypt epithelium. However, reversal of the IL-1β induced gut epithelial damage by *Lactobacillus plantarum* clearly indicates the existence of synergistic host-microbiota interactions during early pathogenic infection and supports the potential role of these mechanisms as targets for therapeutic intervention.

Lee et al. [82] also showed the ability of Paneth and stromal cells to detect the lactate produced by Lactic Acid Bacteria (LAB) microbiome members through the Gpr81 receptor and promote ISCs proliferation and epithelial regeneration via Wnt3 signaling.

This initial evidence highlights that the secretory functions of Paneth cells that take place in the ISC crypt are crucial for the regulation of the intestinal microbiota through the direct detection of the intestinal commensal bacteria. However, the role of Paneth cells as critical regulators at the interface between microbiota and ISCs compartment deserves further exploration.

### 4.5. ISCs Regulation Mediated by Microbiota-Induced Production of microRNA

Interestingly, new evidence has been provided on microbiota control of ISCs proliferation through microRNAs (miRNAs) changes. Intense scientific research has indeed shown that miRNAs are key molecular regulators of various biological functions, including stemness features related to colorectal CSCs, also in response to environmental stimuli [36]. Moreover, it has been demonstrated that different miRNAomes can be found in functionally distinct cell types of the intestinal epithelium [83,84] and that miRNAs respond to the microbiota in a highly cell-type-specific manner [85]. ISC miRNA levels have been found to be particularly sensitive to the microbiota due to direct influence by bacteria residing within the ISCs crypt or to changes in the microenvironment, such as metabolites or endotoxins release or immune cells activation [86]. Among ISC miRNAs regulated by gut microbiota, miR-375 knockdown has been reported to increase ISCs proliferative capacity [87]. 

In addition, small changes in ISC miRNAs expression may result in an alteration of the composition of intestinal epithelial cells. Accordingly, the manipulation of a single miRNA (miR-30) has been shown to promote enterocyte differentiation [88], and it is conceivable that other as yet unknown miRNAs may influence the fate of different cell types belonging to the intestinal crypt, including goblet and Paneth cells, thus ultimately affecting the secretory functions of the crypt epithelium with consequences on the microbiota composition. 

It should be noted that in the gut, extracellular vesicles loaded with miRNAs can be secreted both basolaterally and apically into the lumen [89]. In a recent study, a possible modulation of gut microbiota by these luminally-located miRNAs has been proposed [90].

It is clear that the relationship between miRNAs and microbiota in influencing gut epithelium homeostasis through ISC crypt modulation is multifaceted, interconnected, and highly complex, but the comprehension of miRNAs dependent host-microbiota interactions is still very limited. Further research is necessary to explore such interesting relationships. This will allow to elucidate a key molecular network that contributes to the control of intestinal homeostasis and that is of considerable interest and of high therapeutic relevance in the treatment of CRC and other gastrointestinal diseases associated with impaired ISCs function. 

## 5. Effects of Dysbiosis on ISC Niche Impairment in CRC

Several metagenomic analyses have reported that the host-microbiota mutually beneficial symbiosis existing under physiologic conditions is lost during a state of pathological microbial imbalance due to alteration of the microbiota composition (dysbiosis) and/or host genetic susceptibility. 

According to the “driver-passenger” model proposed by Tjalsma et al. [91], the dysbiosis at the origin of CRC may initially be caused by the colonization of driver bacteria with procarcinogenic features that may potentially initiate CRC development. This results in a gradual change in the tumor microenvironment, where a secondary colonization of passenger bacteria can cause further transformation of the epithelial phenotype into hyperplasia, and adenoma into carcinoma.

Many studies have relied on the identification of the microbial species associated with CRC, as thoroughly reviewed by Ternes et al. [92], and have shed light on the possibility that bacteria interfere with the molecular mechanisms underlying CRC. Metagenomic analyses and functional studies in animal models are progressively identifying the roles of different bacteria in CRC evolution, including *Fusobacterium nucleatum* and some strains of *Escherichia coli* and *Bacteroides fragilis* [93,94,95]. These findings could be extremely useful for clinical applications, such as the identification of the gut microbiota biomarkers with diagnostic, prognostic or predictive significance, or intestinal modulation to prevent cancer or enhance the effect of the specific therapies.

**Figure 2:**
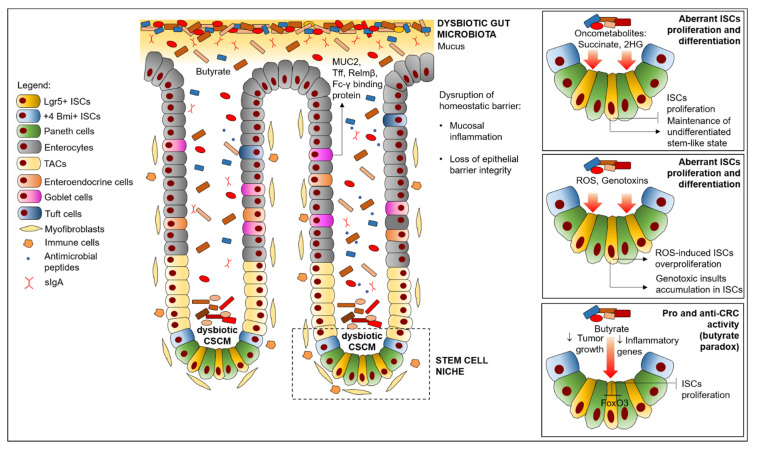
Effects of dysbiosis on ISC niche impairment in CRC. Abbreviations: CSCM, crypt-specific core microbiota; ISCs, intestinal stem cells; ROS, Reactive Oxygen Species, sIgA, secreted immunoglobulin A; TAC, transient-amplifying cells; 2HG, L-2-hydroxyglutarate.

However, despite the many roles that the dysbiotic communities may play in CRC development are still an object of intense research efforts, here we primarily focus on the most recent findings that link the gut microbiome to CRC pathogenesis through ISC niche impairment. In particular, we will describe how a dysbiotic condition can directly or indirectly affect ISC or its associated microenvironment (ISC niche) by different means, thus providing a series of stimuli that finally predispose the host organism to CRC development (Figure 2).

### 5.1. Microbiota Promotion of Colon Inflammation, Leading to ISC Niche Impairment in CRC

In recent years, many studies have shown that the inflammatory microenvironment contributes to the process of tumor initiation and progression, including CRC pathogenesis [96]. Since the ISC niche has a close connection with the inflammatory microenvironment, we can assume that a frequent tumorigenic mechanism is promoted by inflammation, through the enhancement of proliferative and survival signaling in the ISC niche. Several lines of evidence indicate that different molecular and cellular mechanisms participate in the reciprocal action between ISCs/CSCs and inflammatory mediators, involving the release of inflammatory cytokines (interferons (IFNs), tumor necrosis factor (TNF), IL-6, IL-17) and the activation of inflammatory cells, such as myeloid-derived suppressor cells and tumor-associated macrophages [97]. Consistently with this, among the protumorigenic inflammation-associated pathways in CRC, the particularly active signaling pathways in the transformed intestinal epithelium are those involved in the commitment and maturation of myeloid and lymphoid cells, such as those associated with NF-κB and signal transducer and activator of transcription 3 (STAT3) [98]. In these conditions, excessive intestinal inflammation due to an imbalance between the gut microbiota and mucosal immunity could represent a critical source of stimuli predisposing to CRC also through ISC niche impairment.

For example, enterotoxigenic *Bacteroides fragilis*, which is abundant in the stool samples of CRC patients [99], induces the expression of proinflammatory chemokines in colonic epithelial cells [94] and is responsible for morphological changes in epithelial cells, mucosa damage, and mucin degradation due to the presence of metalloprotease and *B. fragilis* toxin (BFT) [100]. In addition, experiments in mice have revealed that *B. fragilis* coordinates a procarcinogenic inflammatory cascade via IL-17-dependent STAT3 and NF-κB signaling in colonic epithelial cells, also supporting the differentiation of myeloid-derived suppressor cells and tumor-associated macrophages [94]. In contrast, *Nontoxigenic B. fragilis* exerts an immunoprotective role against colitis-associated CRC, which is mediated by TLR2 signaling in mice [101].

Another recent research paper reported that *Enterococcus faecalis* can cause colitis after infection and, in murine intestinal epithelial cells, can induce TGF-β expression, thereby activating the Smad signaling pathway [102]. Under physiological conditions, TGF-β down-regulates inflammatory responses to commensal bacteria and helps to induce immune tolerance. Interestingly, it has been strongly suggested that TGF-β signal transduction drives dedifferentiation and enhances stem cell properties in CRC [103]. In addition, *E. faecalis* leads to the expression of progenitor and tumor stem cell markers. As a further inflammation-related tumorigenic mechanism, it polarizes colon macrophages to produce endogenous mutagens that initiate chromosomal instability (CIN), and drives CRC through a bystander effect. This provide new insights into the mechanism underlying CRC initiation based on endogenous transformation and tumor stem cell marker expression through a microbiome-driven bystander effect [104].

Such emerging evidence highlights the significant role of the crosstalk between CSCs and the inflammatory microenvironment associated with a dysbiotic condition in CRC initiation and progression. 

### 5.2. Production of Microbiota Metabolites Affecting ISCs Proliferation/Differentiation in CRC

Following the first evidence obtained in *Drosophila*, showing the ability of the commensal *Acetobacter pomorum* to regulate host energy metabolism and ISCs activity [105], a growing body of research supported the hypothesis that a dysbiotic condition can affect human niche stem cells’ functions in terms of metabolic dysregulation. 

Several studies have established the importance of the microbiota in CRC initiation through the alteration of gut microbial homoeostasis and the disruption of the physiological secretome/metabolome in the intestinal microecosystem based on a subtle equilibrium between normal cells and beneficial microbes. In this section, we describe the various classes of metabolites and secretory products that are released into the tumor microenvironment by the gut microbiota. We discuss the effects of the microbial products on the ISC niche through the modulation of the host metabolism, a topic still in its infancy, and highlight the contribution of ageing and dietary intake to the modulation of gut metabolites’ production and function.

The oncometabolites-gut microbiota metabolome includes growth factors, cytokines, proteases, kinases, but also metabolic intermediates that have been seen to progressively accumulate in cancer, either upstream (e.g., L-2-hydroxyglutarate, succinate, fumarate) or downstream (e.g., D-2-hydroxyglutarate, lactate) of metabolic defects [106]. A variety of these metabolites are closely related to CRC [107]. For example, *B. fragilis* and some *Prevotellaceae,* but also *F. nucleatum* and *E. coli* produce succinate, which exerts a proinflammatory role [108]. Usually, succinate in the gut lumen is an intermediate of the propionate production in several species such as *Veilonella parvula* and *Phascolarctobacterium succinatutens* [109]. Succinate is an interesting metabolite produced by both the microbiome and the host and its accumulation produces several effects on both of them [108]. For example, succinate produced by LPS-activated macrophages acts in a paracrine and autocrine way to promote IL-1β secretion [110] and sustains inflammation. Lumen succinate accumulation is sensed by enterohemorrhagic *E. coli* (EHEC) and activates the expressions of virulence genes [111] that are able to reduce the mucus layer [112]. Interestingly, succinate was found to affect ISCs activity by suppressing cell proliferation [113] inducing superoxide production and mucosa damage in the colon [114].

Another example is the metabolic intermediate L-2-hydroxyglutarate (2HG) produced by the *E. coli* catabolism of lysine to succinate, which is involved in epigenetic deregulation and reprogrammed metabolism in numerous types of cancer [115]. Consequently, the 2HG putative oncometabolite could stimulate carcinogenesis by keeping malignant cells in an undifferentiated stemlike state [116]. Moreover, it has been proposed that 2HG overproduction may take part in a metabolic dysregulation involving disruption of mitochondrial pyruvate metabolism in intestinal epithelial cells, resulting in a significant effect on ISC proliferation, according to recent studies in *Drosophila* [117].

Furthermore, lactate, which is one of the main products of the symbiotic LAB, usefully applied in therapeutic approaches through the regulation of lactate in the tumor microenvironment, seems to sustain cancer cells in particular conditions and is actively exchanged among glycolytic and oxidative tumor cells [118]. As mentioned above, microbial lactate is sensed by Paneth and stromal cells and promotes ISCs proliferation as observed after the administration of two LAB, such as *Bifidobacterium* and *Lactobacillus* spp., in mice. Microbial lactate is sensed by the Grp91 receptor on stromal and Paneth cells and through Wnt3 and PORCN signaling promotes ISCs proliferation and differentiation [82].

Short-chain fatty acids (SCFA)-Saccharolytic fermentation stimulated by a high-fiber diet produces SCFAs, namely butyrate, acetate, and propionate. The mechanism of SCFAs absorption can be mediated by passive diffusion or more often by dedicated transporters on the intestinal epithelial cells [119]. It has been suggested that in a condition of “leaky” gut permeability due to tissue damage or senescence, SFCAs exert their metabolic regulations on the host stem cells contributing to the accumulation of mitochondrial damage accompanied by the imbalance between glycolysis and oxidative phosphorylation. This induces an abnormal accumulation of ROS and eventually aberrant stem cell proliferation/differentiation and, in turn, depletion of stem cells [120].

At the cellular level, SCFAs can affect processes such as cell proliferation, differentiation, and gene expression, either directly or indirectly. In particular, butyrate, which is produced by specific colonic bacteria, predominantly *Clostridia* clusters XIVa and IV of *Firmicutes* [121], can drive the epigenetic regulation of gene expression through the inhibition of histone deacetylases (HDACs) and the activation of histone acetyltransferases (HATs) [122]. Butyrate can also regulate some miRNAs, such as miR-106b [123] and miR-92a [124], which regulate p21 and p57 gene expression in CRC, respectively. 

Regarding the effects of SCFAs on ISCs, a proliferation-promoting role on intestinal epithelial cells has been indicated [125]. However, there are conflicting data on this topic, possibly due to the existence of unmanageable confounders in different studies, which may contribute to diverging results, for example the lack of standardized animal feed. Furthermore, it should not be excluded that SCFAs exert opposite effects on ISCs through different mechanisms. For instance, butyrate is used by colonocytes as their main energy source [126] and, consistently with this, it can stimulate ISCs proliferation. On the other hand, butyrate can affect the expression of genes regulating proliferation that may or may not support ISCs proliferation, as observed in vivo [3] and in vitro in small intestinal organoids [127]. Paralleling this dualistic role on ISCs, there are no concordant versions on the tumor suppressive role of butyrate in CRC, as highlighted by the “Butyrate Paradox” [128].

In addition to influencing ISCs proliferation, SCFAs have been proven to induce differentiation to goblet cells, as demonstrated in vitro and in germ-free mice monocolonized by *Bacteroides thetaiotaomicron*, a gut microbiota species known to produce acetate [129]. Further in vitro studies based on human intestinal cell lines or mouse small intestinal organoids also revealed the ability of propionate to enhance the expression of the genes involved in goblet cell differentiation via PPAR-γ signaling [130].

Mounting evidence suggests that SCFAs can be linked to a lower risk of CRC and successfully used for CRC prevention or therapy. For example, butyrate suppresses proinflammatory genes and tumor growth and inhibits the proliferation of healthy ISCs [3,131]. However, the mechanisms underlying a possible role for SCFAs in reprogramming ISCs fate during colorectal tumorigenesis are still unclear. Further research on the mechanisms of butyrate-induced pro- and anti-CRC activities will be crucial for the prevention of CRC by improving diet regimens.

Secondary bile acids—increased levels of bile acid in the gut, for example due to a high-fat diet [132]—seem to favor Gram-positive members of the *Firmicutes* [133]. Among them, some bacteria 7α-dehydroxylate host primary bile acids to toxic secondary bile acids, including deoxycholic (DCA) and lithocholic (LCA) acids. Both DCA and LCA can significantly induce CRC development by multiple molecular mechanisms involving ROS production, DNA damage, and chromosomal instability [134]. Interestingly, these secondary bile acids were found to promote the generation of colorectal CSCs [135,136].

Reactive Oxygen Species (ROS) microbes produce free radicals that damage DNA. For instance, *Enterococcus faecalis* can produce extracellular superoxide and hydrogen peroxide, which can cause DNA damage in colonic epithelial cells [137]. Similarly, *Bacteroides fragilis* produces ROS which can damage host DNA and contribute to CRC [138]. *Peptostreptococcus anaerobius* also induces ROS accumulation, an event that supports cholesterol biosynthesis, which in turn induces cell proliferation and causes dysplasia in mice [139]. *P. anaerobius* is significantly enriched in the feces and tissues of CRC patients [140].

Overall, although the precise regulation of stem cell activity in response to the high levels of ROS produced by microbial species remains obscure, ROS overproduction has been clearly associated with different types of tissue damage, and, in particular, oxidative stress has proved crucial to prompt ISCs overproliferation and CRC initiation [141].

Bacteria implicated in CRC are characterized by the expression of genotoxins, which can directly alter the gene pools in target cells, resulting in genomic instability and ultimately triggering cancer development. This can be observed, for example, in response to the different types of genotoxins produced by *E. coli*. Among them, polyketide synthase-positive *E. coli* produces colibactin, a genotoxin that induces DNA double-strand breaks in host cell DNA both in vitro and in vivo studies [142,143]. Cytotoxic necrotizing factor 1 (CNF1) is another protein toxin produced by extraintestinal pathogenic *E. coli*, which in vitro can block cell mitosis and enhance endoreplication, thus inducing cell multinucleation, polyploidy and ultimately genomic instability in the progeny [144]. Recent works have documented that *E. coli* stimulates tumor formation both in vitro and in Familial adenomatous polyposis (FAP) patients in the presence of enterotoxigenic *Bacteroides fragilis* co-colonization [100,145].

How microbiota-dependent genotoxins affect many of the survival and proliferation pathways of ISCs is beyond being completely understood. Further studies in this direction will help to elucidate the extent to which genotoxic insults derived from the gut microbiota can contribute to the formation of CSCs, which are intrinsically tolerant to DNA damage and fail to undergo cell death upon the accumulation of genetic and epigenetic alterations.

### 5.3. Invasive Microbes Inducing ISCs Impairment in CRC

Under normal conditions, the intestinal mucosal lining forms an effective barrier against invading pathogens and provides a suitable environment for commensal microbes to outcompete potentially pathogenic bacteria. The commensal microbiota is metabolically active against pathogens, causing starvation of their competitors, and through this it indirectly plays a key role in maintaining the mucosal layers and epithelial integrity [146,147].

In dysbiosis, which can be due to genetics, environmental factors, diet, drug intake [148], pathogenic strains can take over the altered commensal microbiota or can cause dysbiosis themselves. What happens during infection by harmful microbes is therefore a local disruption of the mucosal lining caused by inflammation, which principally occurs as a first-line immune reaction against a pathogen [149]. In this context, different mechanisms can be responsible for tissue damage. A tissue can be directly injured by pathogens or be attacked by immune cells that respond to pathogens, such as activated ROS-producing immune cells, finally leading to the invasion of damaged tissue by harmful microbes. Tumor sites have impaired intestinal epithelial barriers that can further enhance carcinogenesis by allowing invading bacteria to penetrate the intestinal mucosa, resulting in increased inflammation [150].

The functional relationship between inflammation-dependent tissue damage and cancer is not new. However, a particular role of the inflammatory microenvironment has been recently proposed as a cancer-inducing niche responsible for the development of CSC [151]. As an extension of this concept, it could be assumed that microbiota-dependent pathogens invasion associated with an inflammatory status could influence, through a complex system of regulators that control the proliferation/differentiation balance of the ISCs, the niche cell turnover and consequently result in altered epithelial barrier function.

Consistent with this, recent work documented the alteration of tumor stem cell function in murine CRC due to intruding bacteria, including *E. coli* and/or *Shigella*, as well as *Citrobacter*. This effect was triggered by activation of TLR-dependent calcineurin/NFAT signaling in tumor cells, resulting in the upregulation of stem cell-associated genes (Cd44v6, Lgr5, Olfm4 and Dclk1) [152].

Similarly, the dysbiotic behavior of constitutively invasive variants of nonpathogenic commensal bacteria was correlated with CRC tumorigenesis. Sahu et al. demonstrated that the aberrant host internalization of *E. coli* induces intestinal CSCs expansion and tumorigenicity by activating multiple host signal transduction cascades downstream of microbe sensing pathways Nod1/Rip2 and TLR/MyD88 [153].

Interestingly, these studies support the idea that microbe-driven tumorigenesis may not only result from a possible commensal virulence, but could be determined by self-derived features of the commensal microbiota at the interface with the host mucosal barrier. In this scenario the biofilm would seem to play a key role in colon carcinogenesis. Dejea at al. [154] defined biofilms as “aggregations of microbial communities encased in a polymeric matrix that adhere to either biological or nonbiological surfaces” and demonstrated, for the first time, the link between bacterial biofilms and CRC. Biofilms, predominantly present in the proximal ascending colon, may create procarcinogenic environments, strictly connected to the accumulation of proinflammatory taxa which can affect the physiological processes of the colorectal epithelium leading to alter the polyamine metabolism with consequent modification of the regulation of inflammation, apoptosis and cell proliferation [155,156]. This effect can also occur at the ISC niche level, exercising a different role in the homeostatic or tumorigenic context. Along these lines, it has been shown that particular microbiota components belonging to biofilm structures organized in the gut mucus layer can invade the colonic crypts. These microbes mainly consist of known right colon cancer (RCC)-associated bacteria [154,157]. It is not excluded that the adherent microbes contained in the crypt-specific core microbiota may represent a crucial link between environmental stimuli and stem cell dysregulation to yield focal lesions. In this regard, they could be considered important mediators of the tumor initiation. At the same time, understanding what drives the formation of biofilms and if this process can be modulated through microbial manipulation techniques, as in the case of the use of engineered bacteria, could represent an interesting starting point for future research activities [158]. Initial encouraging results in animal models have shown indeed that the engineered *Escherichia coli Nissle 1917* expressing biofilm-disrupting enzyme is capable of counteracting *Pseudomonas aeruginosa* biofilm infection [159], an effect that has been demonstrated similarly by probiotic bacteria, *Lactobacilli*, against the biofilm of *Vibrio cholerae*, an intestinal pathogen [160]. 

## 6. OMICS Approaches at the Host-Microbiota Interface toward Novel Precision Medicine Strategies in CRC

### 6.1. Omics Approaches Potential in Deciphering Host-microbiota Interface at ISC Niche Level

Recent developments in High Throughput Sequencing technologies (HTS) and bioinformatics tools applied to the study of microbial communities associated with a specific habitat, have allowed for much deeper knowledge than was previously possible. What is present, what is expressed, what is translated, what is produced are just some of the questions that “omics” approaches can answer.

Significant advances in “omics” technologies applied to the study of microbiota in the form of (meta)genomics, (meta)transcriptomics and (meta)proteomics, as well as epigenomics and metabolomics, have provided a large volume of information regarding the identification of taxa, gene functions, biological pathways, microbial evolution and metabolic networks. 

It should be pointed out that the study of microbiota, both in physiological and pathological conditions, such as CRC, is influenced by numerous technical variables as well as by the type of sample from which it is isolated. Fecal samples are frequently used in human gut microbiome studies, with results consistently reporting enrichment of *Peptostreptococcus stomatis*, *Parvimonas micra*, *Porphyromonas* spp. and *Fusobacterium nucleatum* in the feces of CRC patients [161,162,163,164]. More meaningful mechanistic data about the host-microbe relationship in CRC can be obtained by analyzing biopsy samples of gut mucosa, due to the adherence of the microbial cells to the intestinal epithelium. Recent studies analyzing this type of sample reported that *Fusobacterium*, *Parvimonas*, *Gemella* and *Leptotrichia* are most significantly enriched in early-stage CRC [165], while *Fusobacteria* and ε-*Proteobacteria* increase as the CRC stage advances [166]. Instead, much, if not all, has yet to be revealed at the ISC niche level. 

However, deciphering the interaction between the microbiota and the intestinal crypt with a higher cell resolution, i.e., at the ISC niche level, is a challenge that could be successfully faced by applying different “omics” approaches.

Among them, Metagenomics is commonly used to reveal microbial diversity and provide an overview of the microbiome species distribution in a sample [167]. Huge advancements in microbial DNA sequencing techniques, represented by the “amplicon-based” and “shotgun” approaches, and the bioinformatics tools required to analyze the data sets obtained, have made it possible to achieve a much more in-depth profiling of the composition of the microbial communities, even at the stem niche level. In particular, the amplicon-based metagenomic approach, targeting two hypervariable regions of the 16S rRNA gene, helped researchers to identify a crypt-specific core microbiota (CSCM) in both human and murine colon [8,62].

Metatranscriptomics, on the other hand, aims to evaluate the expression level of microbial or host genes, also in response to specific stimuli, by applying of a complex technique consisting in the synthesis of complementary DNA (cDNA) from genes transcribed in the messenger RNA (mRNA) then sequenced. In the case of gut microbiome, Metatranscriptomics provides a true systems biology approach to identify microbiome reactions to environmental changes, such as exposure to antibiotics, pathogenic infection or probiotic administration. The RNAseq analysis was applied by Peck et al. (2017) [86] to investigate the factors and mechanisms that influence the interaction between microbiota and ISC. In this study, the microbiota appears to exert control of ISC proliferation in part through miRNA-dependent regulation. In the presence of the microbiota, miR-375 was found to be suppressed and these data correlated with an increased proliferative capacity of ISC. The applied approach also allowed them to demonstrate that the miRNA profiles are cell-type specific in the intestinal epithelium and their expression levels also change in relation to the gut microbiota, ensuring the control of the function and overall homeostasis of the intestinal epithelium.

Understanding the function and interactions of the gut microbiota in their entirety is the goal of Metaproteomics and Metabolomics. The first approach provides information of the functioning microbial proteins. Characterization of the metaproteome, using 2D-PAGE and mass spectrometry, is expected to provide data linking genetic and functional diversity of microbial communities. It holds significant promise for cancer microbiome research. For example, proteomic technologies can aid in the exploration of the effects of the gut microbiota on the pharmacodynamics of cancer therapy drugs [168]. It can also provide new insights into microbial components with potential anticancer function, as observed for *E. coli* and *Staphylococcus aureus* which release outer membrane vesicles (OMVs) containing trypsin-sensitive surface proteins. These vesicles can reportedly induce anticancer cytokines in a mouse model of CRC, resulting in a significant reduction in tumor burden [169]. On the other hand, Metabolomics enables the identification and quantification of metabolites deriving from an altered metabolism in response to pathophysiological stimuli [170]. Powerful spectroscopic techniques, such as nuclear magnetic resonance (NMR) spectroscopy and/or mass spectrometry, are recognized as the most powerful techniques used in Metabolomics. In the context of cancer microbiome, different metabolites can derive from the host metabolism, microbial metabolism or cometabolism between microbiota and host. A successful Metabolomics approach was used to demonstrate that succinate accumulation in presence of a dysbiotic microbiome, as shown in IBD patients, is probably due to loss of succinate-consuming microbes (*Bacteroidetes* and *Negativicutes*) [108]. Hence, Metabolomics provides a valuable tool for elucidating functional interactions between dysbiosis and changes in host physiology, including the development of cancer and intestinal inflammatory diseases [171,172]. The evaluation of the gut microbiota metabolic activity may also be a predictor of chemotherapeutic toxicity in CRC [173,174]. Another interesting field of Metabolomics application is “pharmacomicrobiomics”, which would allow us to understand the host-microbiota crosstalk between the metabolic and immune functions in a host with or at risk of cancer, in response to therapy [175]. To the best of our knowledge, there are still no published data regarding the application of Metabolomics to investigate the interaction between microbiome and ISC, an aspect that could become an interesting research focus for future studies.

While large sequencing endeavors are focused on exploring the whole genome of all microbes in the environment, single-cell sequencing approaches may be employed to analyze the least abundant microbial species within community samples [176]. In fact, natural heterogeneity is averaged in traditional sequencing approaches, whereas single-cell sequencing can potentially recognize cellular differences within heterogeneous cell populations in any tissue or cell culture. For instance, single-cell RNAseq was used to define the proportions of different intestinal cell types and their responses to bacterial and helminth infections such as *Salmonella* and *Heligmosomoides polygyrus*. In this study, the ISCs gene expression profiles revealed that the response to different infections follows similar (activation of stress gene modules) but also specific patterns resulting in restructuring of the epithelial barrier [177]. Single-cell sequencing has already achieved other important results, such as the discovery of bacteria with an alternative genetic code [178], the characterization of specific bacterial species isolated from mouse gut microbiome samples [160], the ability to observe which gut microbial components are foraged by host-compounds [179], and the absolute quantification of the gut microbiome taxon abundances [180,181]. 

The isolation of cells from solid samples such as swabs, biopsies and tissues, and the analysis of very low starting quantities of DNA and RNA, sometimes down to femtograms of material, represents a relevant limitation for single-cell sequencing techniques. Along with these aspects, it is also important not to underestimate the technical difficulties associated with the bioinformatic analysis of data. In fact, new challenges arise in bioinformatics and biostatistics to cope with the exponential increase in studies exploiting single-cell sequencing. In scRNA-Seq the methods of quality control, normalization, differential gene calling, and clustering are different from those applied in traditional bulk sequencing [182]. In microbiome investigation, Single Amplified Genomes (SAG) sequencing allows access to the genome of single microbes, but new bioinformatic workflows need to be developed to address low-coverage issues [183]. 

However, this technology shows significant advantages over other approaches, such as Metagenomics. It can not only generate a high-quality genome for minor microbial community members, but can also identify the functions of individuals within the community, linking these functions to specific species. Moreover, such a powerful technology can simultaneously analyze the microbial genomes and extrachromosomal genetic materials in a cell, thus evaluating host-microbe interactions at cell level [184,185], a property that could be very useful if applied to the exploration of the microbiota-ISC niche interaction.

### 6.2. Application of Different Multiomics to CRC Microbiota Research towards Precision Medicine

With advances in HTS approaches and bioinformatics tools, microbiome studies have expanded beyond simply profiling of microbiota composition, integrating multiomics analyses for a more comprehensive assessment of microbial communities. This strategy integrates the ability of Metagenomics to achieve the taxonomic composition in a microbial community along with the analysis of Metatranscriptome, Metaproteome, and Metabolome, leading to unravel the possible functional implications of changes occurring in a complex microbiota. 

Successful results were achieved when assessing the role of multiomics data analysis in gut microbiome and host-microbe interactions [186] or food-microbial interactions [187]. 

An example of how the integrative multiomics approach can increase the understanding of ISCs biology is clearly shown in the paper by Habowski et al. [188]. They developed a cell-sorting protocol to discriminate between different cellular types and applied single-cell Transcriptomics and Proteomics in mice to develop a model of stemness loss along cell differentiation. Interestingly, the changes in expression profiles from ISCs to committed precursors were principally characterized by splicing events rather than by genes switching off. Moreover, the intersection between transcriptomic and proteomics data revealed that although the levels of mRNAs encoding proliferation markers (e.g., Mki67, Pcna, and Mcms) are higher in stem cells than in differentiated ones, their protein products are still detectable in differentiated cells.

As far as the application of the multiomics approach to the study of microbiome influence on the ISC niche homeostasis, a recent study also including lipidomic and metabolomic analyses evaluated the diet-microbiome-host interactions using mouse intestinal organoids [189]. This study illustrated the potential of multiomics to provide valuable new insights into the mechanisms by which the ISC niche is affected by nutrient-gene or microbiome-gut epithelium interactions. This could open up new opportunities to exploit specific microorganisms in personalized nutritional strategies, as well as for a better understanding of the role of gut microbiota in the initiation, development and possibly prevention of CRC. 

However, the application of integrative omics approaches to the study of host-microbiota interactions at the ISC niche level could be complicated by the heterogeneity among the different niche cell types with different biological processes continuously occurring at the single cell level. Consequently, omics approaches are progressively focusing on the study of individual cells [190,191]. Thus, emerging single cell-based strategies could pave the way for future analyses describing the influence of microbiota on the distribution of cell types, transcriptional profiling, as well as the proteome and metabolome at the single cell level.

The multiomics strategy poses new challenges in both biostatistics and bioinformatics to develop methods and tools able to face and overcome some known limitations associated with the “single” omics (e.g., compositional nature of metabarcoding data in the “amplicon-based” Metagenomics approach) [192]. In this context, an interesting tool is the integrative network where multiomics data are used to produce a snapshot of the crosstalk between the microbes and the host. A pioneering approach was applied by Llyod-Price et al. [193] where ten different data types (e.g., metagenomic, metatranscriptomic, metabolomic, host transcriptomic data) were analyzed to shed light on the IBD pathogenesis.

Integrated multiomics approaches will be gradually included in the framework of precision medicine. In fact, it is now well known that variations in responses to cancer therapy are not only caused by genetic differences between patients [194,195], but also by interindividual differences in gut microbiomes [196,197,198]. It should be noted indeed that some anticancer drugs can induce dysbiosis [199]. This implies the opportunity to improve anticancer treatments by taking into account microbiome-informed patient stratification, through personalized therapies and/or through an adequate manipulation of patient gut microbiota through diet, probiotic and prebiotic interventions, or fecal transplant as tested in recurrent *Clostridium difficile* infection [200].

## 7. Concluding Remarks and Future Perspectives

Intestinal homeostasis is driven by normal ISCs and is deeply influenced by luminal microbiota. In CRC, the exact interaction mechanisms between microbiota and aberrantly reprogrammed CSCs are still unknown. However, the integrative multiomics approach is rapidly evolving and our understanding of the role of gut microbiota-ISCs interaction in colorectal tumorigenesis is expanding.

At the same time, the shift from observational microbiota investigation to association studies, which elucidate the role of specific microbes in modifying the luminal microenvironment and promoting ISCs transformation, might serve to achieve a better understanding of the CRC pathogenesis, as well as to develop novel diagnostic tools and new therapeutic approaches, which might include more direct therapeutic targeting of the pathways that direct ISC differentiation, such as microbiota engineering, TLR targeting, or organoid transplantation. In particular, a future application of the new personalized therapeutic strategies based on microbiota modification could be directed to hopefully prevent/correct the aberrant ISCs reprogramming in the context of dysbiosis linked to the CRC pathogenesis.
